# Vascular sinonasal lesions. How useful is radiology in the diagnosis of rare lesions?^☆^

**DOI:** 10.1016/j.bjorl.2016.04.009

**Published:** 2016-05-18

**Authors:** Abdul Wadood Mohammed, Pearl Sara George

**Affiliations:** KIMS Al Shifa Healthcare Pvt Ltd, Department of Otolaryngology, Kerala, India

## Introduction

Sinonasal lesions have a wide range of presentations and etiology. Vascular lesions of the nose and sinuses usually present as epistaxis. Bleeding nasal lesions are a challenge as they very often present in the emergency. Since biopsy from a bleeding lesion is difficult and may lead to catastrophe, the diagnosis mainly depends on the radiology. Computerized tomography is the mainstay of sinonasal radiology followed by magnetic resonance imaging, whenever required. However, computerized tomography and magnetic resonance imaging would give an accurate picture of the extent of the disease, its usefulness decreases when a proper diagnosis is required. However, radiology would be helpful in differentiating between inflammatory, benign and malignant lesion.^1^ This is particularly important to decide the further plan of action. But, in certain situations, these diagnostic clues tend to be totally misleading. We discuss three cases where the radiological diagnosis and postoperative histopathological diagnosis were entirely different causing practical difficulties in patient management.

## Case description

### Case report 1

**Figure 1:**
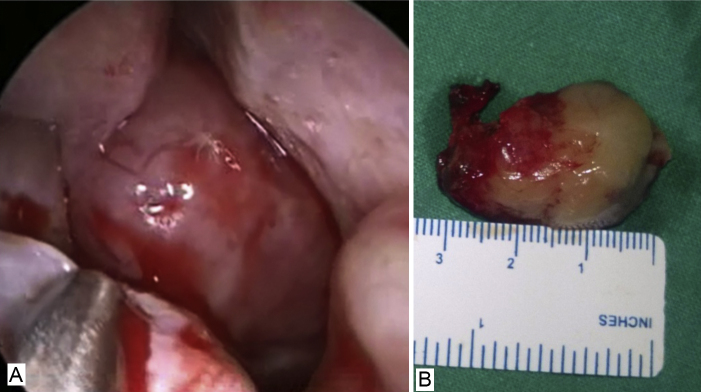
(A) Bleeding mass in the nasal cavity. (B) Mass after endoscopic excision.

**Figure 2:**
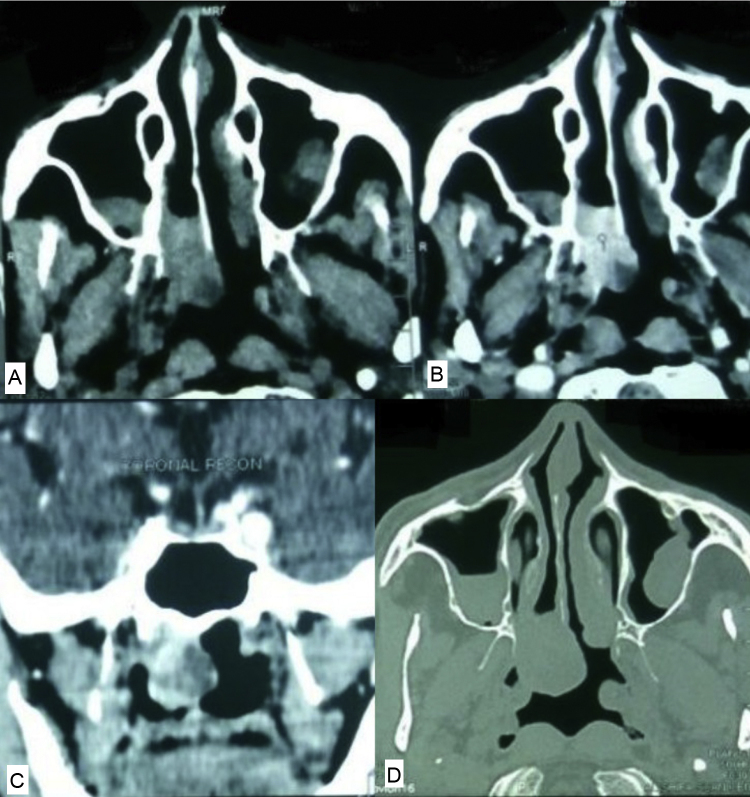
(A) Non contrast computerized tomography. (B) Contrast enhanced computerized tomography-axial. (C) Contrast enhanced computerized tomography – coronal view. (D) Computerized tomography – bone window showing no bone involvement.

An 85 year-old male patient presented to the ENT outpatient department with a history of right sided nasal obstruction of 2 months duration and intermittent right sided epistaxis for a 1 month duration. The bleeding episodes were mild and did not require any consultation or intervention as they stopped spontaneously. He was a known hypertensive on medication. There were no other comorbidities. Nasal endoscopy of the patient revealed a pinkish bleeding mass in the posterior part of the right nasal cavity and nasopharynx (Fig. 1A and B). A contrast-enhanced computerized tomography was done which showed an enhancing well-circumscribed soft tissue lesion in the right nasopharynx and nasal cavity. The lesion seemed to be attached to the lateral wall of nasopharynx (Fig. 2A and B). There was no evidence of bone erosion or extension into any sinus or pterygomaxillary fissure. A primary diagnosis of pyogenic granuloma or lobular capillary hemangioma was made. The differential diagnosis included hemangioma, angiomatous polyp, hemangiopericytoma, and paraganglioma. The patient underwent transnasal endoscopic excision of the tumor. The post-operative histopathology came as plasmacytoma extraosseous.

### Case report 2

**Figure 3:**
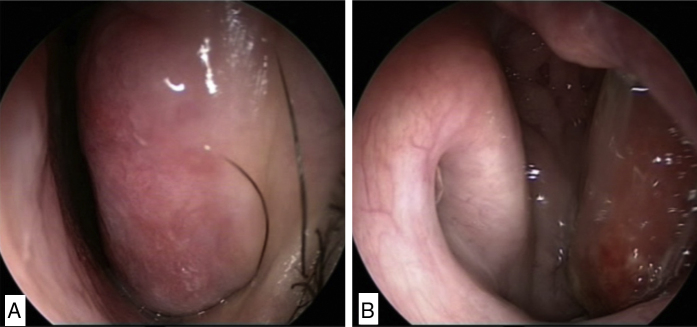
(A) Enlarged inferior turbinate in left nasal cavity. (B) Mass going to the oropharynx from the left nasal cavity.

**Figure 4:**
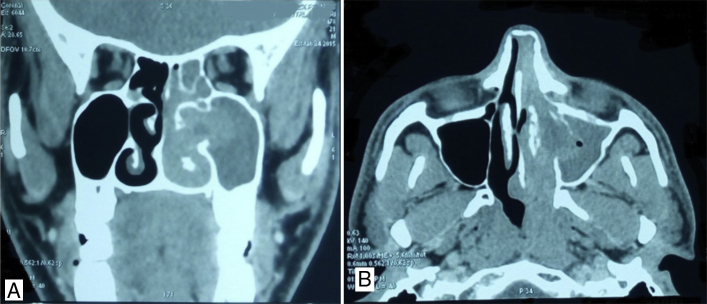
(A) Contrast enhanced computerized tomography – coronal view showing mass filling the left nasal cavity destroying the inferior and middle turbinates. (B) Contrast enhanced computerized tomography – axial view, showing destruction of inferior turbinate along its entire length.

A 22 year-old male presented to our outpatient department with gradually progressing nasal obstruction of the left side for 2 months and swelling over the nasal dorsum and nasofacial groove for 1 month. He also complained of a lumpy feel in the throat and increased watering from the left eye. He gave a history of intermittent oral and nasal bleeding which was trivial and did not require any intervention. On examination, his left inferior and middle turbinates were enlarged and the mass was going posteriorly into the nasopharynx and oropharynx (Fig. 3A and B). There was a diffuse swelling over the left side of nasal dorsum obliterating the left nasofacial groove. Oral cavity examination revealed a reddish mass in the left oropharynx, which was firmly adherent to the posterior pharyngeal wall. On performing the syringing test, the left nasolacrimal duct was found to be obstructed. A contrast-enhanced computerized tomography was done, which revealed a heterogeneously enhancing soft tissue mass involving the left nasal cavity, nasopharynx, and oropharynx. The swelling seemed to arise from the inferior turbinate and middle turbinate destroying both the bones (Fig. 4A and B). Keeping the radiological findings in mind and the age of the patient, the possibility of malignant lesion like chondrosarcoma was considered as a primary diagnosis. Other differential diagnosis included inverted papilloma, esthesioneuroblastoma and lymphoma. Giant cell reparative granuloma, lymphoma and was also kept as a possibility among benign lesions. The patient underwent combined nasal endoscopic and transoral excision with an endoscopic dacryocystorhinostomy. The post-operative histopathology report came as entomophthoromycosis. The patient was given oral itraconazole 100 mg twice daily for 6 months.

### Case report 3

**Figure 5:**
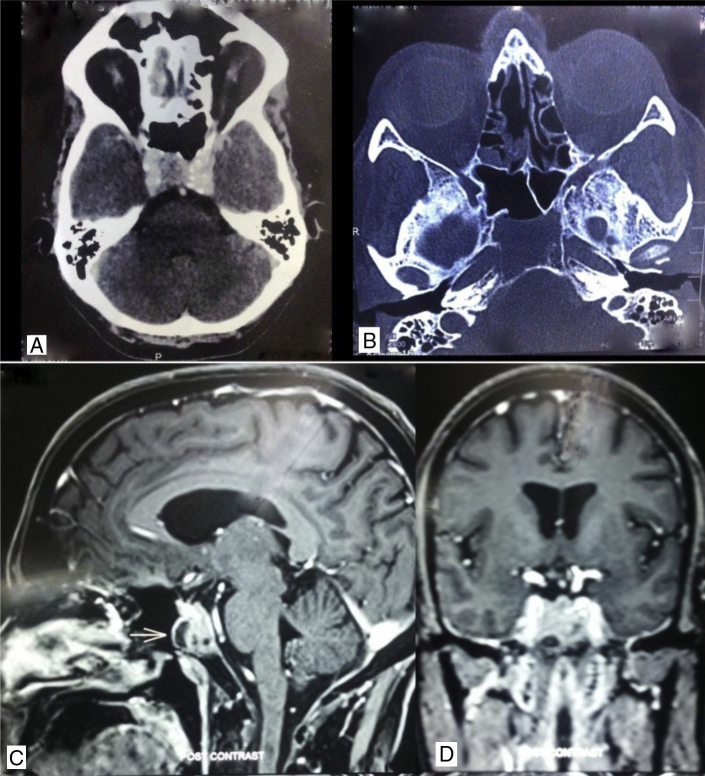
(A) Contrast enhanced computerized tomography axial view showing brilliantly enhancing mass involving the clivus. (B) Computerized tomography – axial view, bone window, showing osteolytic lesion of clivus. (C) Magnetic resonance imaging – sagittal view showing clival lesion with hypoechoic area. (D) Magnetic resonance imaging – coronal view.

A 61 year-old male patient presented to the outpatient department with a history of recurrent early morning nasal bleeding, typically on bending down. He was hypertensive and had coronary artery disease for which he was on anti-hypertensives and antiplatelets in the form of aspirin and clopidogrel. A diagnostic nasal endoscopy was done which showed bleeding medial to the middle turbinate. A contrast-enhanced computerized tomography was done to look for any vascular malformation. The radiology revealed an osteolytic lesion involving the floor of the sphenoid sinus and the clivus. It showed homogenous post contrast enhancement involving the upper clivus without involving the brain and paranasal sinus (Fig. 5A and B). A Gadolinium-enhanced magnetic resonance imaging was done which showed intense enhancement of the lesion with hypoechoic areas suggesting necrosis (Fig. 5C and D). A diagnosis of clival chordoma was made from the radiology. The radiological differential diagnosis included chondrosarcoma, plasmacytoma and nasopharyngeal carcinoma. The patient was taken up for endonasal transsphenoidal clival biopsy under general anesthesia. The post-operative histopathology report was poorly differentiated adenocarcinoma. The patient underwent whole body PET imaging but failed to show any primary lesion. The patient was subjected to radiotherapy.

## Discussion

Sinonasal diseases are very common ranging from inflammatory conditions to malignancies. Since the region is surrounded by bone, computerized tomography remains the first line of investigation, which is further complimented by magnetic resonance imaging. Histopathological examination is required for the final confirmatory diagnosis as most of the tumors have nonspecific radiological features. But radiology is helpful in differentiating the benign and malignant tumors. Features like bone remodeling and thickening are features of benign lesions, whereas bone destruction, ill-defined margins, and the presence of lymphadenopathy are features of malignancy.^1^ However, obtaining tissue for biopsy in vascular lesions, which present with nasal and oral bleeding would further aggravate the condition. Hence, in such situations, the surgeon prefers to reach a provisional diagnosis from the radiological findings to plan for further management. However, in many situations, especially when dealing with rarer diseases the treatment plan and prognosis entirely changes. This may cause many practical difficulties in dealing with the patient.

Our first patient had a vascular lesion in the nasal cavity, which looked benign. The computerized tomography revealed a post contrast heterogeneously enhancing mass without any bony destruction. The lesion was diagnosed as hemangioma due to benign nature and intense post contrast enhancement without any local bone invasion or destruction.^2^, ^3^ The patient was advised complete excision as is the treatment of benign sinonasal lesions. The histopathology came as extramedullary plasmacytoma, which is variant of multiple myelomas. The solitary extramedullary plasmacytoma is an extremely rare variant comprising only 2% of plasma cell dyscrasias.^4^ Biochemical clues of plasma cell dyscrasia are only found in 25% and hence cannot be relied on.^5^ On computerized tomography, they present as mildly heterogeneous, soft-tissue-attenuating masses with moderate-to-marked contrast enhancement with underlying bone destruction. However, in our patient bone destruction was absent which made the diagnosis of malignant lesion impossible. Moreover, there were well-defined margins and no cervical lymphadenopathy. This also caused practical issues in convincing the patient to undergo post-operative radiotherapy to complete the treatment. Since the radiology did not show a possibility of malignancy, the patient was not counseled regarding the need for post-operative radiotherapy, which he eventually declined.

Our second patient had a huge lesion completely filling the entire left nasal cavity. Both the inferior and middle turbinate was involved with the disease and the tumor extending into the oropharynx. The contrast enhanced computerized tomography showed moderately enhancing infiltrating lesion in the left nasal cavity with the destruction of the medial wall of maxillary sinus and inferior and middle turbinate. In sinonasal imaging, generally bone destruction suggests malignancy.^6^ The nasal lacrimal duct involvement also suggested infiltrative lesion. Hence, the possibility of an inverted papilloma, esthesioneuroblastoma, lymphoma, and chondrosarcoma was considered which are malignant neoplasms common in young patients. The histopathological diagnosis was entomophthoromycosis. The possibility of an invasive fungal disease was not considered as the patient was immunocompetant and inflammatory processes produce bone thickening rather than destruction. The radiological finding of entomophthoromycosis is not well discussed in the literature. Rhino facial zygomycosis usually is seen in radiology as mucosal thickening obliterating the nasal air passages and not destroying bone.^7^ They are seen in immunocompetent individuals as opposed to other invasive fungal disease. However, in our patient, there was destruction of the middle and inferior turbinate. Giant cell reparative granulomas present as heterogeneous soft-tissue attenuating masses with homogeneous contrast enhancement on computerized tomography. They also present as expansile lesions with adjacent bone remodeling, lytic bone destruction, and intralesional mineralization.^8^ Lymphoma can present as diffuse infiltrative lesions or masses on computerized tomography destroying the adjacent bones. The patient unnecessarily had to undergo the stress of being diagnosed with malignancy when actually the patient had a relatively benign disease. The patient was advised to take oral itraconazole for 6 months. With an accurate preoperative diagnosis, if it was a smaller lesion, the patient could be even treated with antifungals without surgery.^9^

Our third patient had a vascular lesion in the clivus. Clival tumors are a rare diagnosis and they usually present with cranial nerve palsies. Clival chordoma and chondrosarcoma are the most common tumors of clivus comprising only 0.1%–0.2% of the overall skull base tumors. Metastasis to clivus is rarer and metastasis in the absence of primary tumor is extremely rare.^10^ Clival metastasis is associated with very poor prognosis. Clivus metastasis is difficult to be diagnosed through radiology, as they do not have a characteristic feature and is very similar to chordomas and chondrosarcomas. The only feature that may distinguish clival metastases from chordoma or chondrosarcoma on magnetic resonance imaging is hypointensity in T2 weighted images because of the higher cellular density and lower cytoplasm/nuclear ratio in metastases.

Clival chordoma presents as a centrally located, well-circumscribed, expansile soft-tissue mass associated with extensive lytic bone destruction. The main tumor is usually hyperattenuating with intratumoral calcifications from either destruction or calcification.^11^ Post contrast the tumor has moderate to marked enhancement. Solitary or multiple low-attenuation areas can be seen representing the myxoid and gelatinous material. On T1 W magnetic resonance images the areas of hemorrhage are seen as hyperintense regions. The tumor may show high signal intensity on T2-weighted images according to the high fluid content of vacuolated cellular components. Heterogeneous hypointensity at T2-weighted suggests calcification, hemorrhage, and a highly proteinaceous mucus pool. Hence, these findings are not very different from metastasis. The only strong indicator suggesting clival metastasis would be a positive primary malignancy elsewhere in the body. However, in our patient, as the primary tumor was occult, the diagnosis could not be reached.

## Conclusion

Diagnosis of sinonasal lesions from the radiology may be accurate in the majority of situations. However, in rare situations, the radiological and histopathological diagnosis may be entirely different. Hence, it is always better to discuss the other possibilities at least under broad terms like benign or malignant to the patient before proceeding with surgical excision so that the patient gets prepared for unexpected outcomes.

## Conflicts of interest

The authors declare no conflicts of interest.
